# Dicer suppresses MMP-2-mediated invasion and VEGFA-induced angiogenesis and serves as a promising prognostic biomarker in human clear cell renal cell carcinoma

**DOI:** 10.18632/oncotarget.12520

**Published:** 2016-10-08

**Authors:** Yan-Su Chen, Fei Meng, Hai-Long Li, Qing-Hua Liu, Ping-Fu Hou, Jin Bai, Jun-Nian Zheng

**Affiliations:** ^1^ Jiangsu Key Laboratory of Biological Cancer Therapy, Xuzhou Medical University, Xuzhou 221002, Jiangsu Province, China; ^2^ School of Public Health, Xuzhou Medical University, Xuzhou 221002, Jiangsu Province, China; ^3^ Department of Obstetrics and Gynecology, Huai’ an First People's Hospital, Nanjing Medical University, Huai’ an 223300, Jiangsu Province, China; ^4^ Department of Pathology, Xuzhou Medical University, Xuzhou 221002, Jiangsu Province, China; ^5^ Jiangsu Center for the Collaboration and Innovation of Cancer Biotherapy, Cancer Institute, Xuzhou Medical University, Xuzhou 221002, Jiangsu Province, China

**Keywords:** dicer, metastasis, angiogenesis, prognostic biomarker, renal cell carcinoma

## Abstract

Dicer, a key component of the microRNA processing machinery, has been reported to exert discrepant prognostic values and biological roles in different types of cancers. Here, we investigated the function and prognostic value of Dicer in clear cell renal cell carcinoma (ccRCC). Using the retrospective ccRCC patients’ cohorts with tissue microarray (TMA), we demonstrated that Dicer expression was significantly down-regulated in ccRCC compared with renal non-tumor tissues, and negatively associated with pN status (*P* = 0.005), pM status (*P* = 0.009) and TNM stage (*P* =0.013). Multivariate Cox proportional hazards regression analyses showed that positive Dicer expression was an independent favorable factor for prognosis of ccRCC patients (hazard ratio (HR) = 0.709, *P* = 0.025 for 5-year overall survival; HR = 0.655, *P* = 0.008 for disease specific survival). Moreover, we found that Dicer decreased the abilities of cell migration, invasion and angiogenesis through suppressing MMP-2 and VEGFA expression. Tumor metastasis model *in vivo* showed much more metastatic nodules of lung in the Dicer knockdown group than the control group via increased MMP-2 expression. Our findings imply that Dicer inhibits ccRCC metastasis and may serve as promising prognostic biomarkers for ccRCC patients.

## INTRODUCTION

Renal cell carcinoma (RCC) accounts for 2% to 3% of all malignancies in adult, with 61560 new cases and 14080 deaths in 2015 in United States [1]. Among all the types of RCC, clear cell RCC (ccRCC) is the most malignant form and responsible for most of deaths [2]. Though significant development has been achieved in the diagnosis and treatment of ccRCC, about one third of new diagnosed patients and 20–40% patients with treatment have appeared metastasis [3]. Once the tumor spread out, the median overall survival of ccRCC will reduced to less than one year [4]. Now, based on patients’ clinicopathological manifestations, it is still hard to predict metastasis and the clinical outcomes of ccRCC patients. Therefore, it is urgent for us to inspect the underlying mechanisms of ccRCC metastasis and to identify the specific biomarkers for predicting metastasis and the clinical outcomes of ccRCC patients.

MicroRNAs, small noncoding RNAs containing about 18–24 nucleotides, function as either potential tumor suppressors or oncogenes in cancer development [5]. Dicer, also known as endoribonuclease Dicer, can cleave the pre-microRNAs into mature microRNAs in the cytoplasm [5]. Several lines of evidences have demonstrated that Dicer expression is significantly down-regulated and associated with poor prognosis in some human cancers, such as ccRCC [6], breast cancer [7], colorectal cancer [8], chronic lymphocytic leukemia [9], hepatocellular carcinoma [10]. In addition, low Dicer expression plays important roles in regulating tumor cell proliferation, migration, invasion and angiogenesis [6, 11]. However, the precise mechanism and value of Dicer in ccRCC progression are still little-known.

In this study, we aimed to investigate the expression of Dicer in ccRCC and its association with the clinical features, 5-year survival and disease specific survival of ccRCC patients. Furthermore, we also explore how Dicer regulates ccRCC cell proliferation, migration, invasion and angiogenesis *in vitro*, metastasis *in vivo* and the possible mechanisms.

## RESULTS

### Dicer expression was reduced in the ccRCC compared with non-tumor tissues

**Figure 1:**
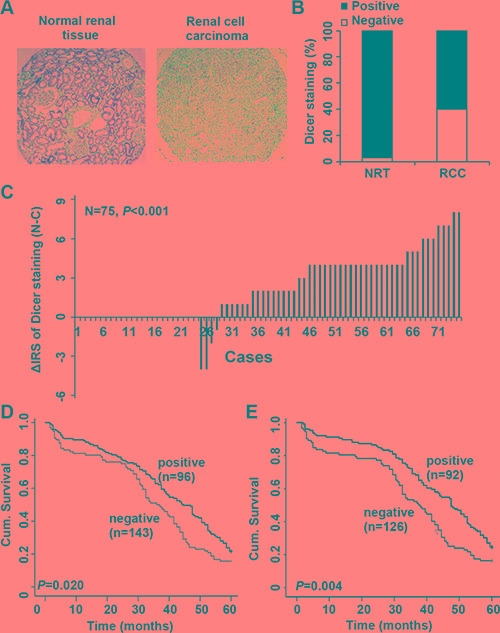
Dicer expression was decreased in ccRCC and positively associated with 5-year overall and disease-specific survival in ccRCC patients Representative images of Dicer immunohistochemical staining in TMA are showed (**A**); Note: original magnification, ×40; NRT: normal renal tissues; RCC, renal cell cancer. The percentages of Dicer positive/negative staining in ccRCC and normal renal tissues (**B**). The distribution of the difference of Dicer staining in ccRCC compared with paired normal tissues (**C**); Note: C: ccRCC tissues, N: paired non-tumor renal tissues, IRS: immunoreactivity score. Kaplan-Meier curves showed the difference of cumulative survival (cum. survival) in 5-year overall survival (**D**) and the disease specific survival (**E**) for the ccRCC patients with positive /negative expression.

To check the expression level of Dicer in ccRCC, TMA containing 295 cases ccRCC tissues and 35 cases normal renal tissues was used to test Dicer protein expressions by IHC. Our data showed that Dicer protein was mainly localized in the cytoplasm (Figure 1A). Positive Dicer expression was explored in 34 out of 35 (97.1%) normal renal tissues and in 177 out of 295 (60%) ccRCC tissues, and had a significant expression difference in ccRCC and normal renal tissues (*P* = 0.002, Figure 1B). Simultaneously, immunohistochemical staining of small TMA was further used to investigate the expression of Dicer in 75 pairs of RCC and adjacent renal tissues. In accordance with our front finding, we also observed a significant decrease of Dicer expression in the cancer compared with the paired renal normal tissues (*P* < 0.001, Figure 1C).

### Dicer expression was associated with clinicopathological characteristics and outcome of ccRCC patients

Fisher's exact test was used to study the association between Dicer expression in cancer and clinicopathological parameters. The data revealed that there were significant negative correlations of Dicer expression with pN status (*P* = 0.005), pM status (*P* = 0.009) and TNM stage (*P* =0.013, Table 1). But we didn’t find any significance between Dicer expression and other clinical features, such as age, gender, tumor size and pT status.

**Table 1:** Dicer staining and clinicopathological characteristics of 295 renal cancer patients

Variables	Dicer staining			
	Negative (%)	Positive (%)	Total	P*
**Age**				
≤ 56 years	58 (41.7)	81 (58.3)	139	0.634
> 56 years	60 (38.5)	96 (61.5)	156	
**Gender**				
Male	70 (35.9)	125 (64.1)	195	0.059
Female	48 (48.0)	52 (52.0)	100	
**Tumor size**				
≤ 7 cm	95 (41.7)	133 (58.3)	228	0.322
> 7 cm	23 (34.3)	44 (65.7)	67	
**pT status**				
pT_1_-pT_2_	94 (39.0)	147 (61.0)	241	0.539
pT_3_-pT_4_	24 (44.4)	30 (55.6)	54	
**pN status**				
N_0_	102 (40.0)	167 (60.0)	269	0.005
N_1_-N_3_	16 (80.0)	4 (20.0)	20	
**pM status**				
M_0_	104 (42.3)	142 (57.7)	246	0.009
M_1_	7 (87.5)	1 (12.5)	8	
**TNM stage**				
I	73 (41.7)	102 (58.3)	175	0.013
II	16 (42.1)	22 (57.9)	38	
III	17 (45.9)	20 (54.1)	37	
IV	10 (83.3)	2 (16.7)	12	

* Two sided Fisher's exact tests.

Some cases were not available for the information.

To determine whether Dicer expression was associated with ccRCC prognosis, the Kaplan-Meier survival curves combined with log-rank test were constructed. Our results demonstrated that ccRCC patients with positive Dicer expression had more favorable 5-year overall survival and disease specific survival than the ones with negative Dicer expression (*P* = 0.020 and *P* = 0.004, respectively; Figure 1D–1E).

Moreover, the univariate and multivariate COX regression analyses continued to be used in detecting whether Dicer expression was an independent prognostic factor in ccRCC. The data of univariate COX regression anaslysis indicated that Dicer expression, tumor size, pT status, pN status, pM status and TNM stage were significantly associated with 5-year overall survival and disease specific survival (Table 2). The multivariate COX regression analysis further showed that positive Dicer expression was an independent favorable prognostic factor for 5-year overall survival and disease specific survival of ccRCC patients after adjusting with classical factors, such as age, tumor size and TNM stage (HR = 0.709, 95% CI = 0.525 to 0.957, *P* = 0.025 for 5-year overall survival; HR = 0.655, 95% CI = 0.479 to 0.896, *P* = 0.008 for disease-free survival; Table 3).

**Table 2:** Univariate Cox proportional regression analysis on 5-year overall and disease specific survival of 295 ccRCC patients

Variable*	Overall survival			Disease-specific survival		
	Hazard ratio	95% CI^†^	*P* *	Hazard ratio	95% CI^†^	*P* *
Dicer						
Low	1.000		0.022	1.000		0.005
High	0.714	0.536–0.952		0.645	0.477–0.873	
Age						
≤ 56 years	1.000		0.637	1.000		0.728
> 56 years	1.069	0.810–1.410		1.053	0.786–1.411	
Tumor size						
≤ 7 cm	1.000		0.003	1.000		0.021
> 7 cm	1.676	1.194–2.352		1.549	1.069–2.244	
pT status						
pT_1_-pT_2_	1.000		0.019	1.000		0.013
pT_3_- pT_4_	1.507	1.071–2.120		1.703	1.469–2.031	
pN status						
pN_0_	1.000		0.002	1.000		0.009
pN_1_- pN_3_	2.982	1.514–5.871		2.599	2.084–3.277	
TNM stage						
I-II	1.000		0.005	1.000		0.004
III-IV	1.647	1.162–2.334		1.742	1.310–2.147	

* *P* values are from Log-rank test.

† CI: confidence interval.

**Table 3:** Multivariate Cox regression analysis on 5-year overall and disease specific survival of 295 ccRCC patients

Variable*	Overall survival			Disease-specific survival		
	Hazard ratio	95% CI^†^	*P*	Hazard ratio	95% CI	*P*
Dicer	0.709	0.525 to 0.957	0.025	0.655	0.479 to 0.896	0.008
Age	1.023	0.760 to 1.376	0.882	1.022	0.749 to 1.394	0.890
Tumor size	1.454	0.993 to 2.129	0.044	1.458	1.274 to 2.183	0.017
TNM stage	1.645	1.210 to 2.123	0.013	1.722	1.284 to 2.208	0.004

* Coding of variables: Dicer was coded as 1 (negative), and 2 (positive). Age was coded as 1 (≤ 56 years), and 2 (> 56 years). Tumor size was coded as 1 (≤ 7 cm), and 2 (> 7 cm). TNM stage was coded as 1 (I-II), and 2 (III-IV).

† CI: confidence interval.

### Dicer inhibited ccRCC cells migration, invasion and angiogenesis *in vitro*

**Figure 2:**
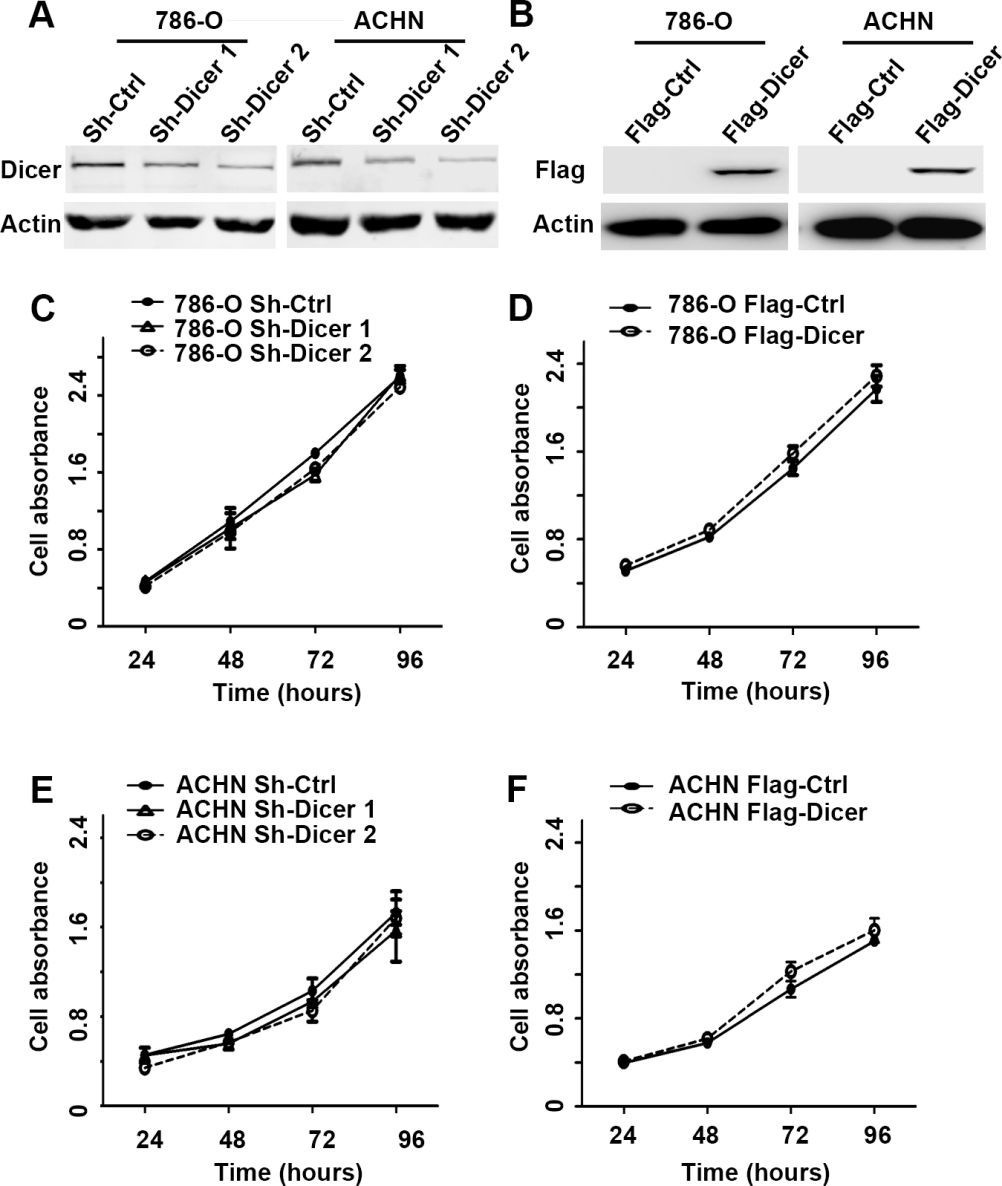
Dicer had no effect on cell proliferation in ccRCC cells *in vitro* Western blot confirmed that Dicer expression in 786-O and ACHN was effectively knocked down or over-expressed by two Dicer shRNA or Dicer expression plasmid when compared with respective controls (**A**–**B**). The CCK8 assays were performed to test the cell proliferation rates of 786-O and ACHN cell with different Dicer expression levels (**C**–**F**). Data were presented as mean ± SD, ^*^P < 0.05, ^**^P < 0.001; Analysis of Variance.

To investigate the role of Dicer in ccRCC progression, 786-O and ACHN cells were stably infected with lentivirus-mediated control shRNA or two effective Dicer shRNAs (Figure 2A), or transiently transfected with Dicer over-expression or control plasmids (Figure 2B). Then the CCK8 assays were performed and the data showed that the cell proliferation rates were similar regardless of Dicer expression levels (Figure 2C–2F).

**Figure 3:**
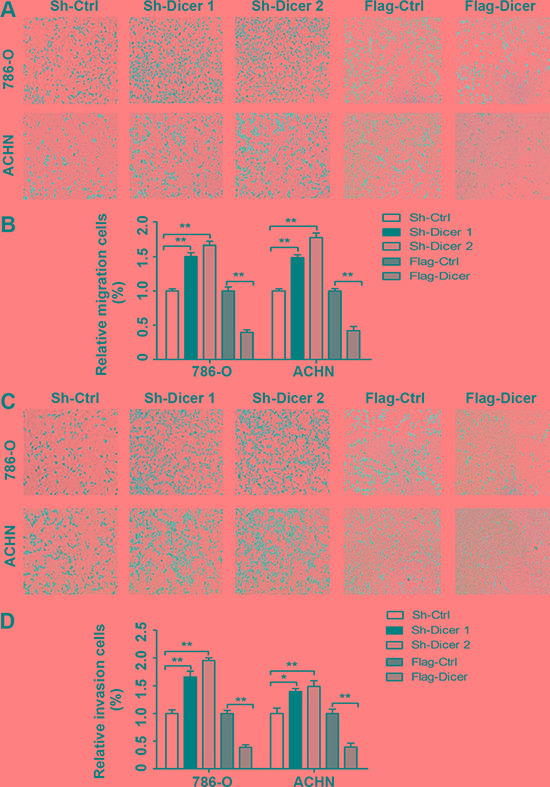
Dicer inhibited ccRCC cell migration and invasion *in vitro* The migration of 786-O and ACHN cell with different Dicer expression levels (**A**) and the number of cell migration per field was counted in five random fields (*n* = 3/group) in 786-O and ACHN (**B**). The invasion of 786-O and ACHN cell with different Dicer expression levels (**C**) and the number of cell invasion per field was counted in five random fields (*n* = 3/group) in 786-O and ACHN (**D**). Data were presented as mean ± SD, ^*^P < 0.05, ^**^P < 0.001; Analysis of Variance.

Since in the ccRCC patient cohort, we found that negative Dicer expression was significantly associated with the positive pN and pM status, we further tested the metastatic function of Dicer in ccRCC cells. The transwell assays were carried out and our data indicated that the abilities of cell migration were significantly increased 1.50 and 1.66 fold in 786-O cells and 1.49 and 1.77 fold in ACHN cells by these two Dicer shRNAs when compared with respective controls; whereas Dicer over-expression decreased cell migration by 60% and 58% in 786-O and ACHN cells when compared with respective controls (Figure 3A–3B). In line with these findings, these two Dicer shRNAs significantly increased the abilities of cell invasion by 1.66- and 1.95-fold in 786-O cells and 1.40- and 1.49-fold in ACHN cells when compared with respective controls, while Dicer over-expression decreased the abilities of cell invasion by 61% and 60% in 786-O and ACHN cells when compared with corresponding controls, respectively (Figure 3C–3D).

**Figure 4:**
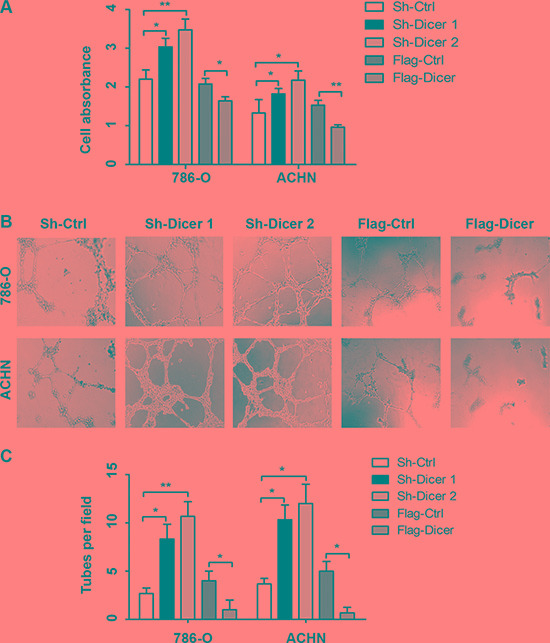
Dicer suppressed HUVECs proliferation and tube formation *in vitro* The HUVECs proliferation in the conditioned medium collected from 786-O and ACHN cells with different Dicer expression levels (**A**). The tube formation by HUVECs in the conditioned medium collected from 786-O and ACHN cells with different Dicer expression levels (**B**). The number of tubes formed per field was counted in five random fields (*n* = 3/group) in 786-O and ACHN (**C**). Data were presented as mean ± SD, ^*^P < 0.05, ^**^P < 0.001; Analysis of Variance.

Since angiogenesis is a key step for solid tumor metastasis [12], here we went on revealing the functional effect of Dicer in ccRCC angiogenesis. We performed HUVECs growth and the tube formation *in vitro*. The growths of HUVECs were significantly accelerated in the conditioned medium from Dicer knocked down 786-O and ACHN cells while significantly slowed in the conditioned medium form Dicer over-expression 786-O and ACHN cells compared with the corresponding controls (Figure 4A). Moreover, the average number of complete tubular structures formed by HUVECs was significantly increased in conditioned medium from Dicer knocked down 786-O and ACHN cells but decreased in that from Dicer over-expression 786-O and ACHN cells when compared with the corresponding controls, respectively (Figure 4B–4C).

**Figure 5:**
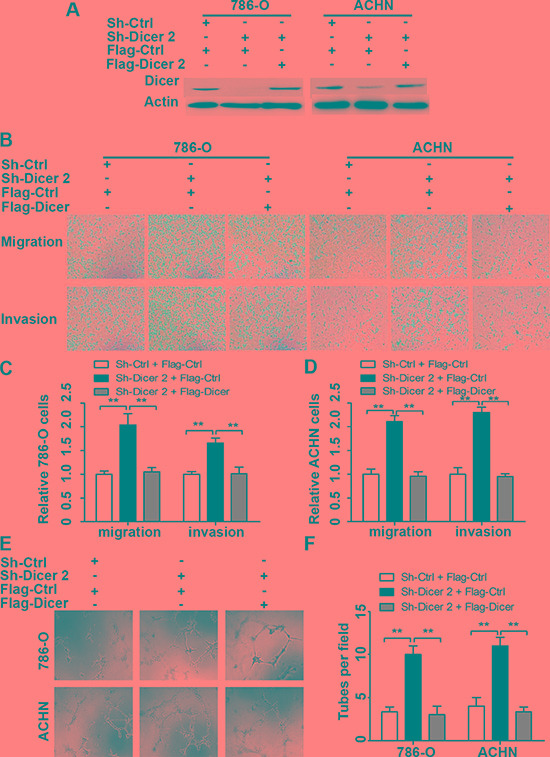
Dicer rescue abrogated Dicer-knockdown-induced ccRCC cell migration, invasion and angiogenesis Western blot confirmed that Dicer expression in Dicer stable knocked down 786-O and ACHN cells was effectively rescued by Dicer expression plasmid when compared with respective controls (**A**). Dicer over-expression in Dicer stable knocked down 786-O and ACHN cells abrogated Dicer knockdown enhanced cell migration and invasion (**B**), the number of cell migration and invasion per field was counted in five random fields (*n* = 3/group) in 786-O and ACHN (**C**–**D**). The increased tube formation by HUVECs in the conditioned medium collected from Dicer stable knocked down 786-O and ACHN cells was abolished by Dicer over-expression (**E**). The number of tubes formed per field was counted in five random fields (*n* = 3/group) in 786-O and ACHN (**F**). Data were presented as mean ± SD, ^*^P < 0.05, ^**^P < 0.001; Analysis of Variance.

In order to verify the role of Dicer in ccRCC cell migration, invasion and angiogenesis, Dicer rescue assays were performed. We over-expressed Dicer in the Dicer stable knockdown 786-O and ACHN cells, respectively (Figure 5A). The transwell assays showed that the increased abilities of cell migration and invasion in Dicer knockdown 786-O and ACHN cells could be significantly inhibited by Dicer over-expression (Figure 5B–5D). Moreover, the elevated tubular structure formations in the conditioned medium collected from Dicer knockdown 786-O and ACHN cells were significantly abrogated in that form Dicer over-expression (Figure 5E–5F).

### Dicer suppressed migration, invasion and angiogenesis of ccRCC cells via inhibiting MMP-2 and VEGFA expression

**Figure 6:**
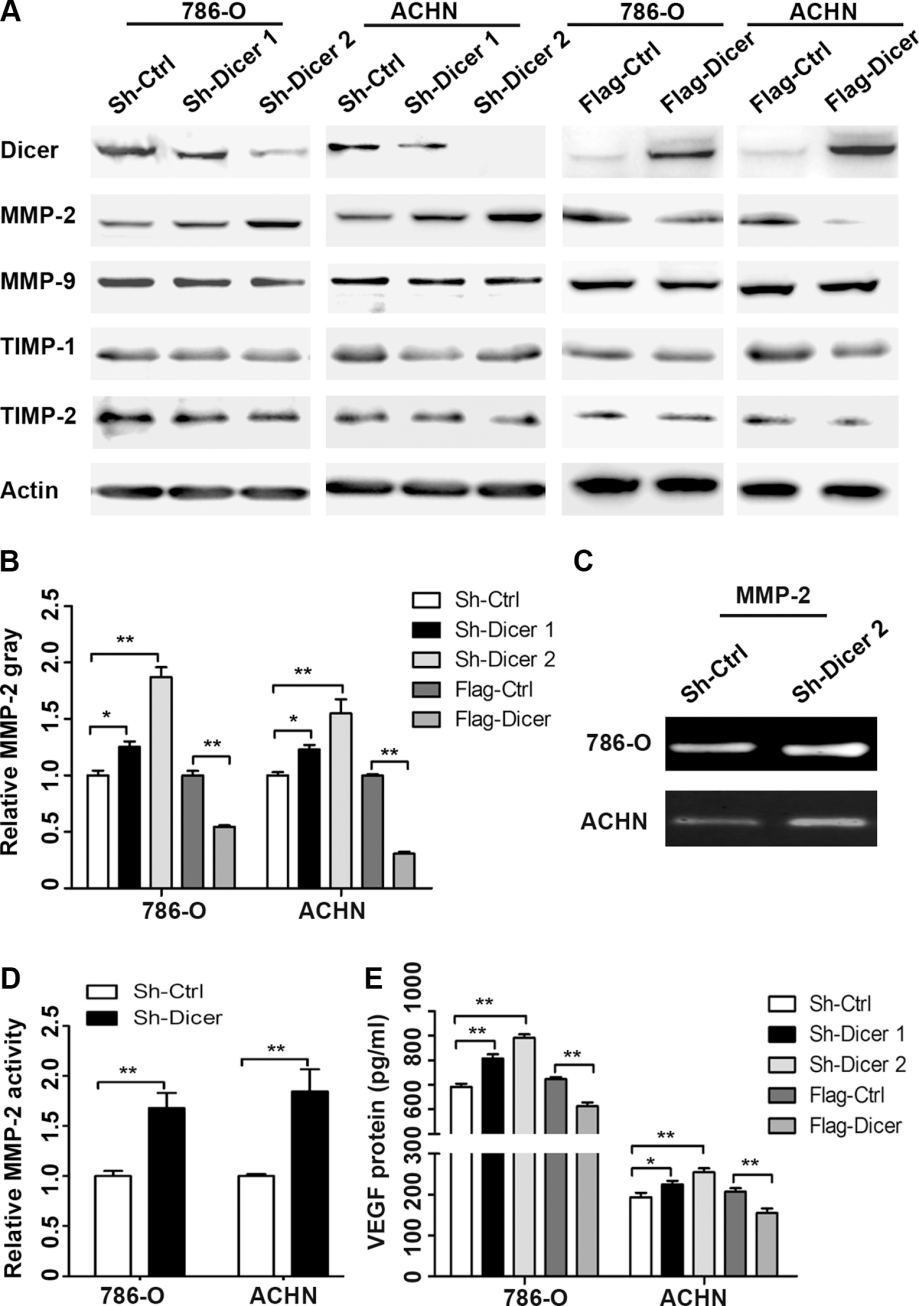
Dicer decreased the expressions of MMP-2 and VEGFA The expressions of MMPs and the inhibitor of MMPs, such as MMP-2, MMP-9, TIMP-2 and TIMP-2 in the 786-O and ACHN cells with different Dicer expression levels were determined by western blot (**A**). The intensity of the MMP-2 protein bands was analyzed by densitometry, after normalization to the corresponding beta-actin level (**B**). Gelatin zymography revealed the enzyme activity of MMP-2 in Dicer knockdown groups and control groups (**C**, **D**). ELISA assay showed the expression levels of secreted VEGFA (pg/ml) in the conditioned medium collected from the 786-O and ACHN cells with different Dicer expression levels (**E**). The data are means ± standard deviations from three independent experiments. Data were presented as mean ± SD, ^*^P < 0.05, ^**^P < 0.001; Analysis of Variance.

To unravel the possible mechanism of Dicer regulated ccRCC metastasis, we investigated the classic molecules expression of tumor metastasis and angiogenesis. As is well-known, matrix metalloproteinases (MMPs) and the tissue inhibitors of MMPs (TIMPs), the most common enzymes in remodeling extracellular matrix components, play important roles in tumor metastasis [13]. Our data showed that Dicer knockdown significantly stimulated while Dicer over-expression significantly suppressed MMP-2 protein expression, but not the expressions of MMP-9, TIMP-1 and TIMP-2 in 786-O and ACHN cells compared with the respective controls (Figure 6A–6B). Simultaneously, the gelatin zymography was used to test the alteration of MMP-2 activity. Our results indicated that MMP-2 activity was significantly increased by Dicer knockdown in 786-O and ACHN cells compared with the respective controls (Figure 6C–6D). Since tumor angiogenesis is a complex process needing multiples of pro-angiogenic factors to take part in [14]; and vascular endothelial growth factors (VEGFs) are the most important triggered factors in stimulating angiogenesis [15]. Our ELISA assays demonstrated that Dicer knockdown significantly promoted while Dicer over-expression significantly inhibited the expression of VEGF in two RCC cells (Figure 6E).

**Figure 7:**
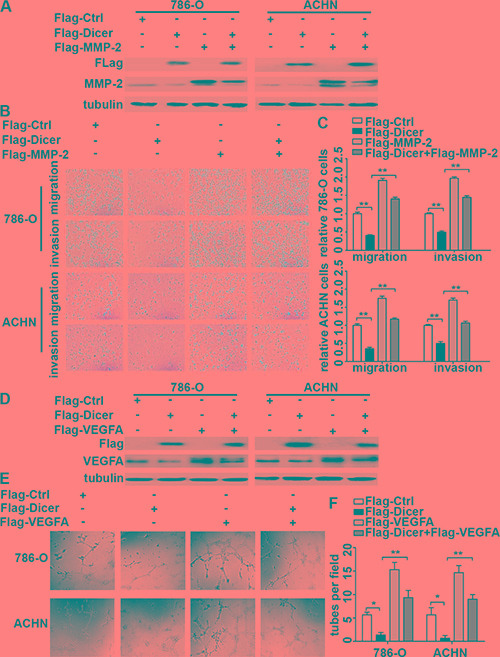
MMP-2 and VEGFA over-expression rescued Dicer-knockdown-increased ccRCC cell migration, invasion and angiogenesis Dicer and MMP-2 were over-expressed separately or in combination in ccRCC cells, and the expressions of Dicer and MMP-2 were confirmed by western blotting (**A**). The migration and invasion assays were performed (**B**), and the number of cell migration and invasion per field was counted in five random fields (*n* = 3/group) in 786-O and ACHN (**C**). Western blot confirmed that Dicer and VEGFA in 786-O and ACHN was effectively over-expressed (**D**) and conditioned medium was collected and applied in HUVEC tube formation (**E**). The numbers of tubes formed per field were counted in five random fields (*n* = 3/group) (**F**). Data were presented as mean ± SD, ^*^P < 0.05, ^**^P < 0.001; Analysis of Variance.

In order to strengthen the conclusions above, 786-O and ACHN cells were transiently transfected with control or Dicer expression plasmid together with or without MMP-2 expression plasmid (Figure 7A), and the results of cell migration and invasion showed that MMP-2 over-expression significantly rescued Dicer-over-expression-decreased cell migration and invasion abilities (Figure7B–7C). Moreover, we over-expressed Dicer and VEGFA in ccRCC cells separately or in combination (Figure 7D), and collected their conditioned media for testing their impact on tube formation of HUVECs. The tube formation assay showed that VEGFA over-expression rescued Dicer-over-expression-inhibited tube formation of HUVECs (Figure 7E–7F). These data suggested that MMP-2 and VEGFA act as the downstream target of Dicer in ccRCC cell migration, invasion and angiogenesis.

### Dicer knockdown accelerates the metastasis of ccRCC cells *in vivo*

**Figure 8:**
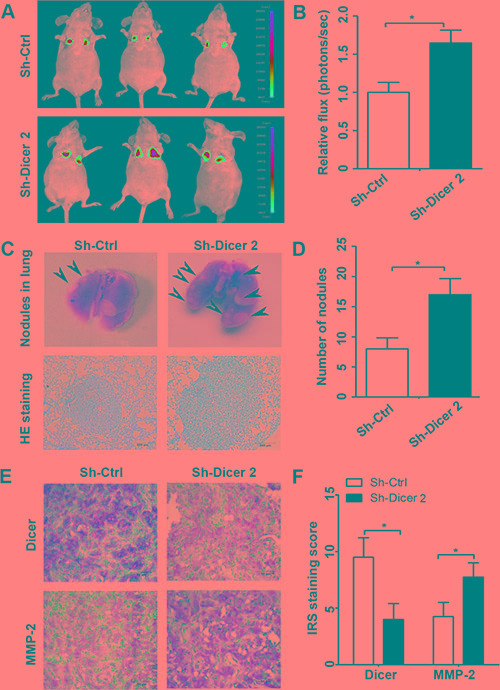
Dicer knockdown accelerated metastasis of ccRCC cells *in vivo* Representative bioluminescence images of mice in the stable Dicer knockdown group and control group (**A**). The bioluminescence imaging tested the Fluc activity in the Dicer knockdown and control groups (**B**). Representative photographs of lung metastasis and H&E staining in the Dicer knockdown and control groups (**C**). The number of metastatic nodules of lung was counted (**D**). The protein expressions of Dicer and MMP-2 in the metastatic nodules were tested by IHC (**E**). The IRS staining scores of Dicer and MMP-2 in the metastatic nodules were evaluated (*n* = 3) (**F**). Data were presented as mean ± SD, ^*^P < 0.05, ^**^P < 0.001; Student's *t* test.

To further confirm the properties of Dicer in regulating ccRCC metastasis, we preformed the tail vein metastasis model in nude mice. The stable Dicer knockdown and control 786-O cells transduced with luciferase lentivirus were intravenously injected into BALB/c nude mice. After two months, bioluminescence imaging was used to monitor the metastatic lesions. Our data exhibited that tumor cells mainly metastasized into lungs and the Fluc activity in Dicer knockdown group appeared significantly higher than the control group (Figure 8A–8B). Moreover, in order to validate that metastasis is derived from tumor cells, we isolated lung tissues and took the visual examination and H&E staining. Our data showed that lungs were infiltrated with the poorly differentiated neoplastic cells, and the number of micrometastases in the Dicer knockdown group was much more than the control (Figure 8C–8D). In addition, we performed immunohistochemical staining on formalin-preserved, paraffin sectioned neoplastic tissues. The results revealed that Dicer expressions were significantly reduced, whereas MMP-2 expressions were significantly up-regulated in the knockdown group when compared with the controls (Figure 8E–8F).

## DISCUSSION

Emerging evidences have indicated that functional genes involved in metastasis can serve as promising biomarkers for the prognosis of ccRCC patients [16]. Dicer, a key component of the microRNA processing machinery, has been reported to exert discrepant prognostic values and biological roles in different types of cancers [6, 17–21]. In this present study, we investigated the precise function and prognostic value of Dicer in ccRCC. Using the retrospective ccRCC patients’ cohorts with TMAs, we demonstrated that Dicer expression was significantly down-regulated in ccRCC compared with renal non-tumor tissues, and negatively associated with metastasis and TNM stage. Furthermore, ccRCC patients with positive Dicer expression had a favorable survival, and Cox proportional hazards regression analyses showed that positive Dicer expression was an independent protective factor for prognosis of ccRCC patients. These findings indicated that Dicer was a prognostic indicator for ccRCC patients and may function as a cancer suppressor gene in ccRCC progression.

Tumor progression is characterized by increased growth speed and metastasis of tumor cells [22]. We showed that Dicer expression didn’t affect ccRCC cell proliferation, which was accordant with our clinical finding that Dicer expression was not associated with tumor diameter. Tumor metastasis is a sequential multi-steps, including angiogenesis, invasion, migration, adhesion and extravasation into the target organ, where, again, induction of angiogenesis and so on [23, 24]. The process of tumor angiogenesis includes endothelial cell proliferation, migration and the formation of blood vessels [25]. Our data demonstrated that Dicer significantly inhibited ccRCC cell migration, invasion and angiogenesis *in vitro*. Therefore, it is not surprising to explain our front finding that negative Dicer expression was associated with increased lymph node and distant metastases, and eventually worse survival of ccRCC patients.

Hypoxia is a hallmark of a wide range of advanced solid tumors [26], and it is well-known that hypoxia-inducible factor (HIF) family and hypoxia-responsive/HIF target genes (*e.g. MMPs and VEGFs*), play important roles in cellular adaptation to hypoxia, angiogenesis, and extracellular matrix remodeling [27, 28]. Studies have showed Dicer protein and some Dicer-dependent microRNAs are key to inhibit the expression and function of HIF-α subunits [29, 30], resulting in up-regulation of HIF downstream target genes, especially MMPs, the most common enzymes in remodeling extracellular matrix components for metastasis [13] and VEGFA, the most important triggered factor in stimulating angiogenesis [15]. Here we determined whether Dicer regulated ccRCC metastasis via MMPs and VEGFA, and interesting found that Dicer significantly suppressed the protein expressions of MMP-2 and VEGFA, which may well explain our migration, invasion and angiogenesis results. The roles of MMP-2 and VEGFA in Dicer-mediated ccRCC cell migration, invasion and angiogenesis were further confirmed by co-over-expression of both Dicer and MMP-2/VEGFA, which rescued Dicer over-expression-decreased cell migration, invasion and angiogenesis. Furthermore, our metastasis model *in vivo* showed that Dicer significantly increased metastatic nodules of lung by up-regulating MMP-2 expression, which validated the findings of experiments *in vitro* and ccRCC patients’ cohort. Therefore, combined with these results and the previous studies, we speculated that Dicer could inhibit ccRCC cell metastasis via Dicer-dependent microRNAs/HIF-α/MMP-2 and VEGFA signal pathway, which needs further study to validate.

However, the limitations of this study should be emphasized here that the selection bias was unavoidable from retrospective studies and the common problems of variations in IHC. Therefore, the biomarker should be validated in larger retrospective and prospective ccRCC cohorts.

In summary, Dicer was significantly reduced in ccRCC when compared with the non-tumor tissues. Negative Dicer expression in ccRCC tissues was significantly correlated with metastasis and a poor survival of patients, which can in part be explained by enhanced MMP-2-mediated invasion and VEGFA-induced angiogenesis. Therefore, these results suggested that Dicer may play as a suppressor in the ccRCC progression and are significant prognostic indicator for ccRCC patients.

## MATERIALS AND METHODS

### Patients and specimens

Two independent ccRCC cohorts containing with TMAs were investigated in this study. A small ccRCC TMA was purchased from Shanghai Xinchao Biotechnology (Shanghai, China), which included 75 pairs of ccRCC tissues and matched non-cancerous tissues. All patients’ informed consents were obtained and institutional approval was approved prior to this study. The large TMA was consisted of 310 ccRCC tissues and 35 normal renal tissues collected from Affiliated Hospital of Xuzhou Medical University between 2005 and 2008, and the clinicopathological features of these patients in this cohort were provided in our previous study [30]. Due to lost samples during antigen retrieval, no tumor cells present in the core, finally 295 ccRCC patients in the large cohort were used to evaluate the correlation of Dicer expression with clinicopathological paramters. All patients’ informed consents were obtained and institutional approval was approved by the Review Board of the Affiliated Hospital of Xuzhou Medical University prior to this study.

### Construction of TMAs and immunohistochemistry (IHC)

The large TMA was constructed by contract service at the National Engineering Center for Biochip (Shanghai, China). Each array dot was punched to 1.5 mm diameter from the paraffin tumor block or the normal renal tissues. The standard protocol for IHC of TMA was used as described previously [30]. The polyclonal rabbit anti-Dicer (1:50, Proteintech, USA) and monoclonal rabbit anti-MMP-2 (1:50, Cell Signaling Technology, MA) were used for primary antibody incubation at 4°C overnight. The slides without primary antibody incubation were used as negative control.

### Assessment of IHC

Two pathologists blinded to the clinical data to evaluate the intensity of immunostaining and the percentage of immunoreactive cells. The intensity of staining was scored as 0 (negative), 1 (weak), 2 (moderate), and 3 (strong); the percentage of immunopositive cells was graded as 1 (0%–25%), 2 (26%–50%), 3 (51%–75%), and 4 (76%–100%). The final semiquantitative immunoreactivity score (IRS) depended on the product of intensity of staining and percentage of immunopositive cells, as reported before [31].

### Animals and cell lines

This experiment *in vivo* was approved by the Animal Care Committee of Xuzhou Medical University. Female BALB/c nude mice, 6–8 weeks old, were purchased from NLARSH China (Shanghai, China), and maintained under specific pathogen-free conditions. Human ccRCC cell lines 786-O and ACHN were obtained from the Shanghai Institute of Biochemistry and Cell Biology, Chinese Academy of Sciences (Shanghai, China). These two cell lines were cultured as described before [30] and incubated in a 37°C humidified incubator with 5% CO_2_.

### Plasmids, stable infection and transient transfections

The pEGFP-C1-Sh-Dicer 1 and pEGFP-C1-Sh-Dicer 2 plasmids (GenePharma, Shanghai, China), pEGFP-C1-Sh-control were generated and confirmed before using by DNA sequencing. Lentivirus was used to pack these plasmids and infected the 786-O and ACHN cells following the manufacturer's protocol. The cells were stably selected with puromycin at a final concentration of 3 μg/ml for 3 weeks. The plasmids of pCMV3-C-Flag-Dicer (Cat: HG11350-CF, Sino Biological Inc, China), pCMV3-C-Flag-MMP-2 (Cat: HG10082-M-F, Sino Biological Inc, China), pCMV3-C-Flag-VEGFA (Cat: HG10008-CF, Sino Biological Inc, China) and pCMV3-C-Flag-control were transiently transfected into cells using Lipofectamine 2000 transfection reagent (Invitrogen, Shanghai, China) following the manufacturer's protocol.

### Western blotting and antibodies

Western blots were carried out as previously reported [30]. The rabbit anti-Dicer (1:1000, Proteintech, USA), anti-MMP-2 (1:1000, Cell Signaling Technology, USA), anti-MMP-9 (1:1000, Cell Signaling Technology, USA), anti-TIMP-1 (1:200, Santa Cruz, USA) and anti-TIMP-2 (1:200, Santa Cruz, USA) were used for primary antibody incubation at 4°C overnight. The mouse anti-β-actin (1:1000, Cell Signaling Technology, USA) was used for the protein loading control. Each blot was repeated at least three times. The intensity of the protein bands were analyzed by densitometry after normalization to the corresponding protein controls.

### Cell proliferation assay

100 μl complete medium including 3 × 10^3^ stable Dicer knockdown 786-O or ACHN cells and the corresponding controls were seeded in 96-well plate and cultured for 24, 48, 72 and 96 hours. At the exact time point, 10 μl cell counting CCK-8 solution (Dojindo Molecular Technology Inc, Shanghai, China) was added to each well and incubated at 37°C for 2 hours, and the cell absorbance was measured using a spectrometer reader at 450 nm.

### Cell migration and invasion assay

The migration and invasion assays were performed as described before [31]. In brief, the transwell filter inserts with a pore size of 8 μm were coated without or with matrigel for the cell migration and invasion assays, respectively. 1 × 10^5^ cells were seeded in serum-free medium in the upper chamber. After 24 hours’ incubation at 37°C, cells in the upper chamber were carefully removed with a cotton swab and the cells that had traversed the membrane were fixed in methanol, stained with Crystal violet (0.04% in water; 100 μl), and counted the permeating cells under the inverted microscope and photographed.

### HUVEC growth and tube formation assay

1 × 10^6^ stable Dicer knockdown and control ccRCC cells suspended by 2 ml fresh serum-free medium were cultured in 60-mm plates for 24 hours, and then conditioned medium was collected. For HUVECs growth assay, 100 μl conditioned medium including 5 × 10^3^ endothelial cells were seeded in 96-well plate and cultured for 24 hours. At the exact time point, the cell proliferation assay was performed. For tube formation assay, the 96-well plate was coated with 50 μl Matrigel ™ (BD Biosciences) and kept at 37°C for 2 hours. 1 × 10^4^ HUVECs were suspended in 100 μl conditioned medium and seeded into the pre-coated 96-well plate and cultured for 24 hours, photos were taken under a microscope, and the complete tubular structures were counted in five random fields.

### Gelatin zymography

Gelatin zymography assay was used to explore MMP-2 activity. 1 × 10^6^ stable Dicer knockdown and control ccRCC cells were seeded in 60-mm plate for 24 hours, and 2 ml conditioned medium was collected and concentrated with Amicon Ultra-4–30k centrifugal filters (Millipore, USA) at 4°C. Then, the standard protocol was used as described previously [30]. At last, Gels were photographed and then quantitatively measured by scanning densitometry.

### ELISA

1 × 10^6^ stable Dicer knockdown and control ccRCC cells were seeded in 60-mm plate for 24 hours, and 2ml conditioned medium was collected. For ELISA assay, the secreted VEGFA expression in the conditioned medium was measured with a human VEGF ELISA kit (eBioscience) following with the manufacturer's instructions.

### Tumor metastasis model *in vivo*

To produce metastasis model *in vivo*, the BALB/c nude mice were randomly divided into two groups consisting of 6 mice each. 2.5 × 10^6^ luciferase lentivirus infected stable Dicer knockdown and control 786-O cells were suspended in 120 μl PBS and injected intravenously through tail vein, respectively. After 2 months, 200 μl fluorescein with the concentration of 15mg/ml and 200 μl 1% pentobarbital sodium were intraperitoneally injected into each mouse, respectively. Then, the standard protocol of bioluminescence imaging was used according to the manufacturer's instructions [32]. After that, the mice were sacrificed and their lungs were isolated and fixed in 10% buffered formalin. The number of metastatic nodules presented on the surface of each set of lungs was counted by visual inspection using a stereoscopic dissecting microscope. The paraffin-embedded tissue sections were used for H&E staining and IHC analysis as described previously [30].

### Statistical analysis

Paired Wilcoxon test was used to test the difference of Dicer staining in tumors and their corresponding non-tumors. Fisher's exact test was used to evaluate the significance of Dicer expression in tumors and normal renal tissues and the association between Dicer expression and clinicopathological parameters. Kaplan-Meier method with a log-rank test was used to ascertain the probability of differences in 5-year survival and disease specific survival between the negative Dicer expression group and the positive expression group. Univariate and multivariate Cox proportional hazards regression analysis were performed to estimate the crude hazard ratios (HRs), adjusted HRs and 95% confidence interval (CI) of HRs. All the statistical analyses were performed by STATA statistical software (version 10.1; StataCorp, College Station, TX). A *P* value of < 0.05 was deemed statistically significant, and all tests were two sided.
